# Major bleeding complications and antithrombotic treatment after isolated surgical bioprosthetic aortic valve replacement

**DOI:** 10.1016/j.ijcha.2025.101861

**Published:** 2026-01-06

**Authors:** Rikhard Björn, Joonas Lehto, Markus Malmberg, Vesa Anttila, Jarmo Gunn, Tuomo Nieminen, Juha E.K. Hartikainen, Fausto Biancari, K.E.Juhani Airaksinen, Tuomas Kiviniemi

**Affiliations:** a Heart Center, Turku University Hospital and University of Turku, POB 52, FI-20521 Turku, Finland; b Päijät-Häme wellbeing services county, Lahti, Finland; c Heart Center, Kuopio University Hospital and University of Eastern Finland, Kuopio, Finland; d Department of Cardiovascular Surgery, Centro Cardiologico Monzino IRCCS, Milan, Italy

**Keywords:** Surgical Aortic Valve Replacement, Bleeding, Ischemic stroke

## Abstract

**Background:**

Despite advancements in surgical techniques and perioperative care, postoperative bleeding and neurological complications remain significant concerns after bioprosthetic surgical aortic valve replacement (SAVR). The present study assessed the incidence of short-term and long-term major bleeding and strokes and their association with antithrombotic treatment after isolated bioprosthetic SAVR.

**Methods:**

The CAREAVR study included 721 patients who underwent isolated bioprosthetic SAVR at four Finnish university hospitals between 2002 and 2014. The day-to-day information on short-term antithrombotic treatment was available from a subgroup including 227 patients.

**Results:**

The median follow-up time was 4.9 (interquartile range 3.0–7.0) years. During the 30-day postoperative period, in the subgroup of 227 patients, 31 (13.7 %) patients experienced a major bleeding event, and 13 (5.7 %) patients a major stroke. A vast majority of the bleedings (80.6 %) occurred within two days after the surgery, and the tail effect of preoperative aspirin was present in 54.8 % of episodes, indicating unintentional antithrombotic effect. During the long-term follow-up (>30 days after the index surgery), major bleeding episodes occurred in 40 (5.5 %) patients, and 47 (6.5 %) patients experienced a major stroke. Overall, 23 (57.5 %) of the patients with major bleeding and 13 (27.7 %) of the patients experiencing major stroke were on OAC during the event.

**Conclusion:**

The incidence of perioperative major bleeding was over two-fold compared to major stroke, the majority occurring during the tail effect of preoperatively used aspirin. During the long-term follow-up, the rates of stroke and major bleeds were similar, and most bleeding episodes occurred while on OAC.

## Highlights

• Perioperative bleeding is twice as common as stroke, mostly linked to ASA use.
• Late discontinuation of ASA may predispose patients to early postoperative bleedings.
• The long-term risk of stroke and bleeding rates are similar, often occurring on OAC.

## Graphical abstract

Early postoperative and late incidence of major stroke and major bleeding after bioprosthetic surgical aortic valve replacement (SAVR). A total of 721 patients who underwent isolated bioprosthetic SAVR were included in the study. The incidence of perioperative major bleeding was over two-fold compared to major stroke, the majority occurring during the tail effect of preoperatively used aspirin. During the long-term follow-up, the rates of stroke and major bleeds were similar, and most bleeding episodes occurred while on OAC.

**Figure:**
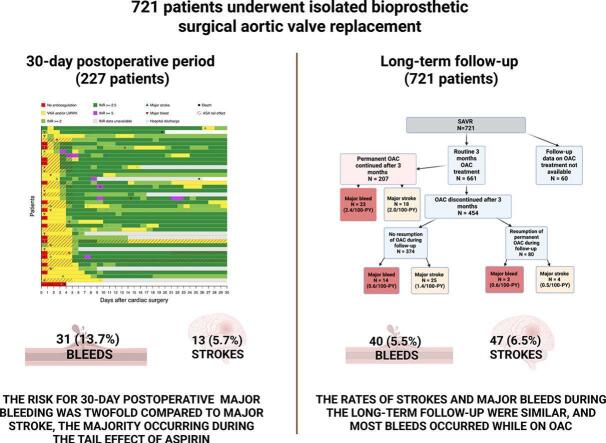

## 1 Introduction

Patients undergoing surgical aortic valve replacement (SAVR) using a bioprosthesis are at elevated early risk of thromboembolic events [1]. Although bioprosthetic valves are less thrombogenic than mechanical prostheses [2], the current European Society of Cardiology (ESC) guidelines recommend that patients with no initial indication for permanent oral anticoagulation (OAC) should be treated with low-dose acetylsalicylic acid (ASA) or vitamin K antagonist (VKA) for the first three months following bioprosthetic SAVR [3]. However, the use of VKA is not without consequence; it has been associated with a significantly increased risk of major bleeding complications [4].

In contrast to bioprosthetic valves, mechanical prostheses necessitate lifelong anticoagulation due to their higher thrombogenic potential. Our previous study highlighted that patients undergoing mechanical SAVR experienced a disproportionately higher incidence of major bleeding events – both in the short and long term – compared to thromboembolic complications such as stroke. Importantly, postoperative bleeding was frequently linked to combined antithrombotic exposure, which included subcutaneous enoxaparin, oral VKA, and the residual effect of recently discontinued ASA, typically persisting for 5 to 7 days [5].

The present study concentrates to the early postoperative bleeding risk after bioprosthetic SAVR, with particular interest on the residual effect of preoperative aspirin. In our patient cohort who underwent bioprosthetic SAVR, prior publications have mainly addressed postpericardiotomy syndrome, stroke and atrial fibrillation (AF), while bleeding complications and the management of antithrombotic therapy have not been thoroughly examined in this patient population [6], [7], [8]. By juxtaposing the incidence of major bleeding against that of major stroke during the early postoperative period, we seek to clarify the risk–benefit profile of current antithrombotic strategies. This analysis is intended to inform clinical decision-making and optimize perioperative management in patients receiving bioprosthetic valves.

## 2 Methods

### 2.1 Study design

This study is part of the Consortium of Studies in the Field of Atrial Fibrillation, Stroke and Bleeding in Patients Undergoing Aortic Valve Replacement (CAREAVR; ClinicalTrials.gov Identifier: NCT02626871), a Finnish multicenter retrospective cohort investigating AF, thromboembolic events, and bleeding complications in patients undergoing isolated SAVR with either bioprosthetic or mechanical valves. The present analysis focuses exclusively on patients who received bioprosthetic valves.

A total of 721 patients who underwent isolated bioprosthetic SAVR between 2002 and 2014 at four Finnish university hospitals were included. Patients were excluded if they underwent concomitant major cardiac procedures, including ascending aortic surgery, coronary artery bypass grafting (CABG), annuloplasty, or left atrial appendage closure. For early postoperative (30-day) antithrombotic treatment analysis, data were available only from one university hospital; the remaining centers were excluded from this subanalysis due to incomplete perioperative treatment records. In addition, routine international normalised ratio (INR) measurements were not available for the control group. A sensitivity analysis comparing baseline characteristics of the subgroup with complete early postoperative data to the rest of the cohort is presented in Supplement Table 1.

All patients were followed long-term, and data on antithrombotic therapy were collected at the time of each endpoint event. Patient records were reviewed individually using a standardized protocol to extract preoperative, perioperative, discharge, and follow-up data, including AF, stroke, transient ischemic attack (TIA), bleeding events, and mortality. Only patients residing within the hospital catchment areas were included to ensure reliable follow-up.

Mortality data were obtained from Statistics Finland, a national registry that tracks causes and timing of death across regions, allowing for extended follow-up even if patients relocated. As such, this resulted in a longer mortality follow-up than the other endpoints.

All patients were given the routine perioperative anticoagulation of enoxaparin 40 mg subcutaneously once daily starting in the evening of the day of the surgery and continuing until VKA treatment (started on the first postoperative day) reached the therapeutic level (INR ≥ 2.0) at all sites. Postoperative antithrombotic management followed a uniform protocol across all participating centers: all patients received only VKA for the first three months after surgery. Preoperative ASA was discontinued on the day of the operation at the latest, and its tail effect was estimated to last 5 to 7 days. The indication for preoperative ASA was mainly primary prevention, and for patients with coronary artery disease, stroke, or TIA, it was for secondary prevention. The routine duration of anticoagulation after surgery was 90 days. In the case of early postoperative AF, the need for permanent OAC after the 90 days was evaluated individually by the treating physician, based on the AF burden and the total risk of ischemic stroke assessed according to Congestive heart failure, Hypertension, Age ≥ 75 years, Diabetes mellitus, Stroke, Vascular disease, Age 65–74 years, Sex category (female) (CHA_2_DS_2_-VASc) score. The primary endpoints of this study were major stroke and bleeding events during the early postoperative period and long-term follow-up. Secondary endpoints were the occurrence of new-onset AF, and all-cause mortality.

During the postoperative 30-day and long-term follow-up, major bleeding was described as an overt, actionable sign of hemorrhage that requires diagnostic studies, hospitalization, or treatment by a health care professional (The Bleeding Academic Research Consortium types 2–5) [9]. “Hospitalization” and “requires diagnostic studies” were excluded from the definition of major bleeding if the patient was already hospitalized. Ischemic stroke was defined as a permanent focal neurological deficit adjudicated by a neurologist and confirmed via computed tomography. Subtypes of ischemic strokes were categorized by treating physicians using the Trial of Org 10,172 in the Acute Stroke Treatment (TOAST) classification system [10]. TIA was defined as a focal neurological deficit lasting less than 24 h and assessed by a neurologist. When a stroke or TIA occurred during the index hospitalization and was diagnosed by the treating physician with confirmation on computed tomography or magnetic resonance imaging, no additional neurological adjudication was required. The study included only ischemic strokes or TIAs considered definite by the treating neurologist or physician. Major stroke was defined according to the TOAST definitions, excluding lacunar strokes (<20 mm in diameter). The diagnosis of AF was confirmed using a 12-lead electrocardiogram (ECG) recording or telemonitoring, indicating an AF episode of 10 min or longer. In all the aforementioned endpoints, only the first endpoint event was included in the analysis, excluding multiple events on the same patient.

Urgent operation was defined as an operation performed during the same in-hospital stay, emergency operation as an operation before the next working day, and salvage procedure in which patients require cardiopulmonary resuscitation en route to the operating theatre or before the induction of anesthesia. Previous cardiac surgery was defined as one or more previous major cardiac operations involving opening the pericardium.

The study protocol was approved by the Medical Ethics Committee of the Hospital District of Southwest Finland and the ethics committee of the National Institute for Health and Welfare (Finland). Because of the retrospective, observational nature of the study, informed consent was not required. The study conformed to the Declaration of Helsinki as revised in 2002.

### 2.2 Statistical analysis

Statistical analyses were performed using the R statistical software version 4.3.2 (R Foundation for Statistical Computing, Vienna, Austria). Continuous variables were reported as mean ± standard deviation for normally distributed data, and as median (25th–75th percentiles) for skewed data. Normality was assessed using the Shapiro-Wilk test and visual inspection. Categorical variables were presented as counts and percentages. To evaluate predictive factors for major complications during the early postoperative 30-day period, Pearson’s χ2 test and Fisher’s exact test were employed. Predictive factors for long-term bleeding risk were assessed using a multivariable mixed-effects Cox regression model, with site number included as a random effect. The model was adjusted for Hypertension, Abnormal Renal/Liver Function, Stroke, Bleeding History or Predisposition, Labile INR, Elderly, Drugs/Alcohol Concomitantly (HAS-BLED) score and age at the time of surgery, considering these as fixed effects. Statistical significance was set at P < 0.05. The proportional hazards assumption was evaluated using Schoenfeld residuals. No correction for multiple testing was applied due to the exploratory nature of the study.

## 3 Results

A total of 721 patients (mean age 75.5 years, 56.4 % female) who had undergone isolated bioprosthetic SAVR were included in the study. The median follow-up time was 4.9 (interquartile range [IQR] 3.0–7.0) years. All 721 patients were included in the long-term follow-up analysis. In addition, day-to-day information on short-term antithrombotic treatment was available from a subgroup of 227 patients, who were included in the postoperative 30-day analysis. The supplementary Table 1 presents baseline characteristics, operative data, and long-term outcomes for the subgroup and the rest of the patient population. Baseline characteristics were balanced between the groups except that patients in the subgroup were slightly older and had more often hypertension and coronary disease.

### 3.1 Early bleeding and stroke events and antithrombotic treatment status

During the 30-day postoperative period, in the subgroup of 227 patients, 31 patients (13.7 %) experienced a major bleeding, and 13 patients (5.7 %) had a major stroke. In addition, in patients with major stroke or bleed, three patients experienced both bleeding event and stroke. The HAS-BLED and CHA_2_DS_2_-VA scores did not differ significantly between patients who experienced major bleeding or stroke events and those who did not. Median HAS-BLED score was 3.0 (IQR 2.0–3.0) among patients with major bleeding and 3.0 (IQR 2.0–3.0) among those without bleeding (p = 0.464). In addition, the median CHA_2_DS_2_-VA score for stroke risk was 2.0 (IQR 2.0–2.0) in patients with stroke and 1.0 (IQR 1.0–2.0) in those without stroke (p = 0.300). Furthermore, three patients (0.4 %) died.

**Fig. 1:**
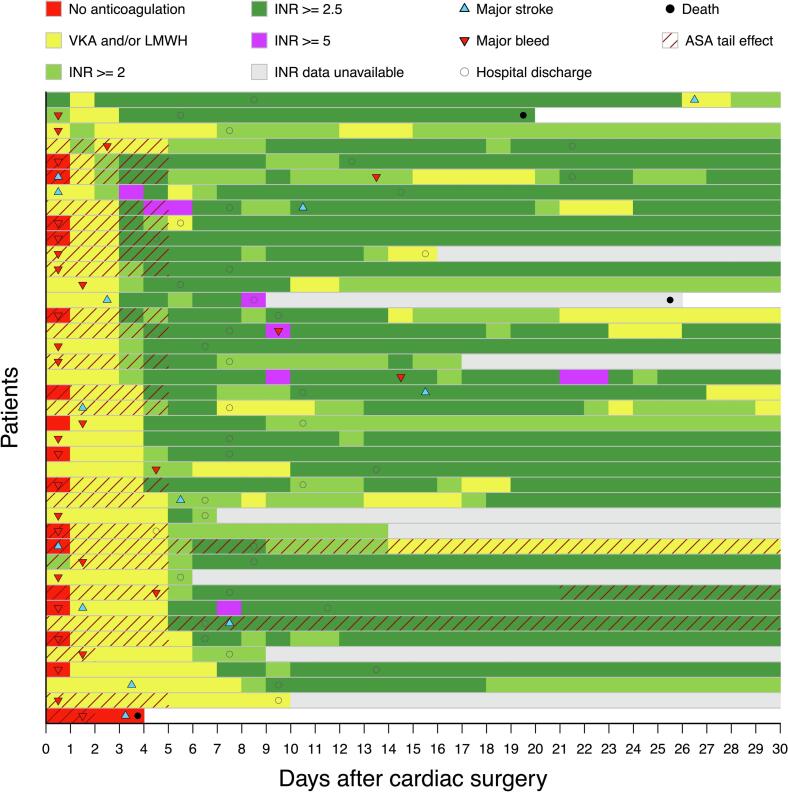
The perioperative antithrombotic treatment of patients experiencing a major stroke or major bleeding during 30 days after bioprosthetic isolated aortic valve replacement. ASA = acetylsalicylic acid; INR = international normalized ratio, LMWH = low molecular weight heparin.

Overall, 138 patients (60.7 %) were on preoperative ASA. These patients demonstrated a higher prevalence of dyslipidemia as well as a greater history of prior stroke or TIA compared with those not receiving preoperative ASA. Detailed baseline characteristics are presented in supplementary Table 2. At the time of major bleeding events, 17 patients (54.8 %) were exposed to combined antithrombotic effects, including subcutaneous enoxaparin, subtherapeutic VKA therapy, and the residual effect of preoperative ASA. Furthermore, 25 out of 31 patients (80.6 %) experienced postoperative bleeding within two days after the surgery. The event rate for major bleeding was 3.4 per 100 patient-weeks in patients with ASA residual effect during the surgery and 2.3 per 100 patient-weeks without the residual effect, during the 30-day postoperative period. The median time to major bleeding was 0 days (IQR 0–1 days), and to major stroke 3 days (IQR 1–7 days). At the time of major bleeding, the median INR was 1.5 (IQR 1.1–2.3), and INR was within the therapeutic range in only 4 (12.9 %) patients. At the time of major stroke, the median INR was 1.3 (IQR 1.0–1.8). The perioperative anticoagulation treatment, timing of adverse events, and baseline characteristics of these patients are presented in Fig. 1 and Table 1. Patients with major bleeding were more frequently using adenosine diphosphate (ADP) receptor inhibitors (p = 0.020) preoperatively. At the time of hospital discharge, they were more likely to be on ASA medication (p = 0.045).

**Table 1:** Baseline characteristics, operative data, and preoperative and perioperative medication of patients experiencing major bleeding or major stroke during the 30-day postoperative period after isolated bioprosthetic aortic valve replacement.

	Major bleed within 30 days, n = 31*	**p1 value**	**Major stroke within 30 days,** n = 13*	**p2 value**	**No major stroke or bleed within 30 days, n = 186**
Age	77.5 ± 3.6	0.615	76.2 ± 3.1	0.307	77.1 ± 3.5
Females	18 (58.1 %)	0.867	8 (61.5 %)	0.720	105 (56.5 %)
Diabetes	2 (6.7 %)	0.113	3 (23.1 %)	0.712	34 (18.3 %)
Dyslipidemia	18 (60.0 %)	0.675	8 (61.5 %)	0.692	104 (55.9 %)
Hypertension	28 (90.3 %)	0.215	12 (92.3 %)	0.470	151 (81.2 %)
Coronary artery disease	13 (41.9 %)	0.911	3 (23.1 %)	0.159	80 (43.0 %)
Atrial fibrillation	5 (16.1 %)	0.270	2 (15.4 %)	0.526	47 (25.3 %)
Chronic atrial fibrillation	1 (3.2 %)	0.139	2 (15.4 %)	1.000	26 (14.0 %)
Paroxysmal atrial fibrillation	4 (12.9 %)	0.764	0 (0.0 %)	0.368	21 (11.3 %)
Chronic lung disease	4 (12.9 %)	1.000	5 (38.5 %)	0.022	23(12.4 %)
Active smoking	2 (8.7 %)	0.665	2 (22.2 %)	0.144	9 (6.7 %)
Active or ex-smoker	6 (27.3 %)	0.262	3 (33.3 %)	1.000	51 (39.9 %)
Body mass index (kg/m2)	26.1 (24.5–27.7)	0.086	27.6 (26.2–29.1)	0.855	27.7 (24.5–30.1)
Active endocarditis	1 (3.2 %)	1.000	0 (0.0 %)	1.000	5 (6.9 %)
Previous endocarditis	1 (3.2 %)	0.372	0 (0.0 %)	1.000	2 (1.1 %)
Previous hepatic cirrhosis	1 (3.2 %)	0.254	0 (0.0 %)	1.000	0 (0.0 %)
Previous heavy alcohol consumption	0 (0.0 %)	1.000	0 (0.0 %)	1.000	2 (2.2 %)
Previous venous thromboembolism	0 (0.0 %)	1.000	0 (0.0 %)	1.000	4 (2.2 %)
Previous stroke or TIA	6 (19.4 %)	0.406	5 (38.5 %)	0.030	25 (13.4 %)
Previous myocardial infarction	2 (6.4 %)	1.000	0 (0.0 %)	1.000	12 (6.5 %)
Previous percutaneous coronary intervention	3 (9.7 %)	0.455	0 (0.0 %)	1.000	12 (6.5 %)
Previous cardiac surgery	1 (3.2 %)	1.000	0 (0.0 %)	1.000	6 (3.2 %)
EuroSCORE II (%)	1.9 (1.4–2.1)	0.982	1.9 (1.5–2.1)	0.978	1.8 (1.4–2.6)
Urgent, emergency or salvage procedure	0 (0.0 %)	1.000	0 (0.0 %)	1.000	6 (3.2 %)
NYHA Class III or more	17 (54.8 %)	0.126	10 (76.9 %)	0.758	128 (68.8 %)
NOAF during index hospitalization	19 (61.3 %)	0.166	5 (38.5 %)	0.512	89 (47.5 %)
Cardioversion during hospitalization	9 (29.0 %)	0.923	3 (23.1 %)	0.759	55 (29.9 %)
Acute de novo dialysis	0 (0.0 %)	1.000	0 (0.0 %)	1.000	1 (0.5 %)
Length of hospital stay (days)	8.0 (7.0–10.6)	0.030	9.0 (8.0–11.0)	0.022	8.0 (6.0–9.0)
**Echocardiographic parameters:**					
Valve prosthesis size (mm)	23.0 (21.0–23.0)	0.744	23.0 (22.0–23.0)	0.990	23.0 (21.2–23.0)
Left ventricular ejection fraction (%)	70.0 (60.0–74.0)	0.008	69.0 (59.5–77.8)	0.071	61.0 (52.0–70.0)
Aortic valve regurgitation	18 (60.0 %)	0.489	8 (61.5 %)	0.765	119 (66.5 %)
Aortic valve peak pressure gradient (mmHg)	90.5 (78.0–106.0)	0.568	84.0 (80.0–96.0)	0.658	88.0 (75.0–102.0)
Mitral valve regurgitation	17 (54.8 %)	0.095	7 (53.8 %)	0.230	126 (70.0 %)
Pulmonary hypertension	10 (40.0 %)	0.920	6 (75.0 %)	0.064	51 (38.9 %)
**Preoperative medication:**					
Warfarin	2 (6.7 %)	0.181	2 (15.4 %)	1.000	33 (17.7 %)
Warfarin interrupted	2 (6.7 %)	0.749	1 (7.7 %)	1.000	21 (11.4 %)
LMWH	0 (0.0 %)	0.603	0 (0.0 %)	1.000	8 (4.3 %)
ASA	20 (60.7 %)	0.501	8 (61.5 %)	0.925	112 (60.2 %)
ADP receptor inhibitor	3 (10.0 %)	0.020	0 (0.0 %)	1.000	2 (1.1 %)
PPI	4 (13.3.%)	0.793	3 (23.1 %)	0.469	31 (16.7 %)
**Perioperative medication:**					
LMWH	29 (93.5 %)	0.055	12 (92.3 %)	0.128	183 (99.5 %)
Warfarin	30 (96.7 %)	0.144	11 (91.7 %)	0.061	184 (100.0 %)
**Discharge drugs:**					
Warfarin	29 (96.7 %)	1.000	12 (100.0 %)	1.000	177 (97.3 %)
LMWH	6 (20.0 %)	0.423	1 (8.3 %)	0.303	49 (26.9 %)
NOAC	0 (0.0 %)	1.000	0 (0.0 %)	1.000	0 (0.0 %)
ASA	1 (3.3 %)	1.000	3 (25.0 %)	0.045	11 (6.0 %)
ADP receptor inhibitor	0 (0.0 %)	1.000	0 (0.0 %)	1.000	0 (0.0 %)
PPI	11 (36.7 %)	0.010	5 (41.7 %)	0.160	41 (22.5 %)

Continuous variables are reported as median (25th – 75th percentiles) or mean ± standard deviation (SD). Values in parentheses are percentages. ADP: Adenosine Diphosphate; ASA: Acetylsalicylic Acid; EuroSCORE: European System for Cardiac Operative Risk Evaluation; INR: International Normalized Ratio; LMWH: Low Molecular Weight Heparin; NOAC: Novel Oral Anticoagulant; NOAF: New Onset Atrial Fibrillation; NYHA: New York Heart Association; PPI: Proton Pump Inhibitor; TIA: transient ischemic attack; P1-value = major bleeding vs no major bleeding or major stroke; P2-value = major stroke vs no major bleeding or major stroke.

*Three patients experienced both major bleeding and stroke and are included in both groups.

### 3.2 Long-term bleeding and stroke events and antithrombotic treatment status

**Fig. 2:**
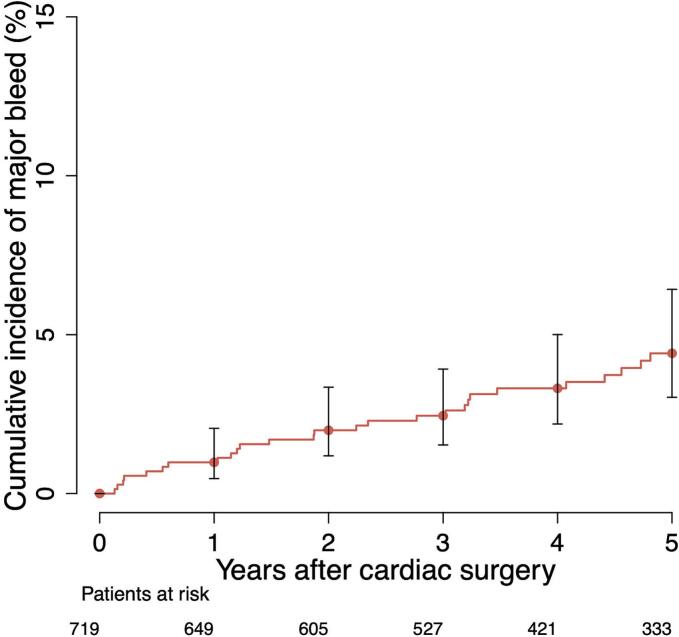
The cumulative incidence of major bleeding during the long-term follow-up after isolated bioprosthetic aortic valve replacement.

**Fig. 3:**
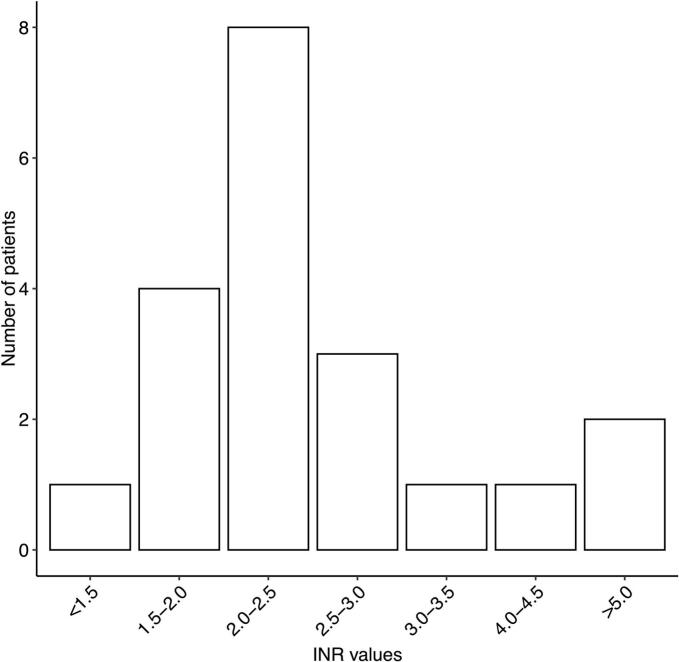
International normalized ratio (INR) values at the time of first major bleeding event during the long-term follow-up in patients on vitamin K antagonist (VKA) therapy. Data on INR values at the time of the event were unavailable for two (0.3%) patients.

**Fig. 4:**
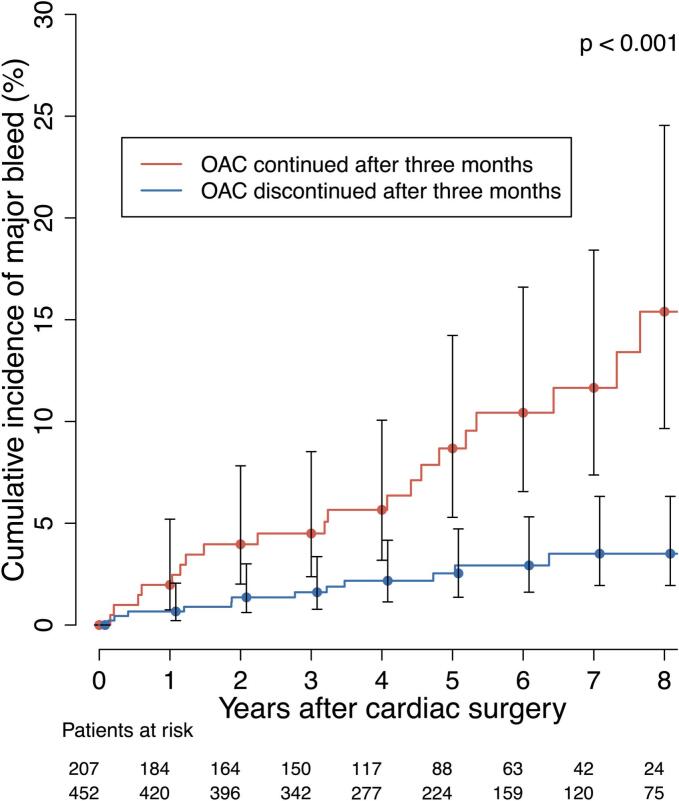
The relation of oral anticoagulation (OAC) on the cumulative incidence of major bleeding during the long-term follow-up after isolated bioprosthetic aortic valve replacement.

**Fig. 5:**
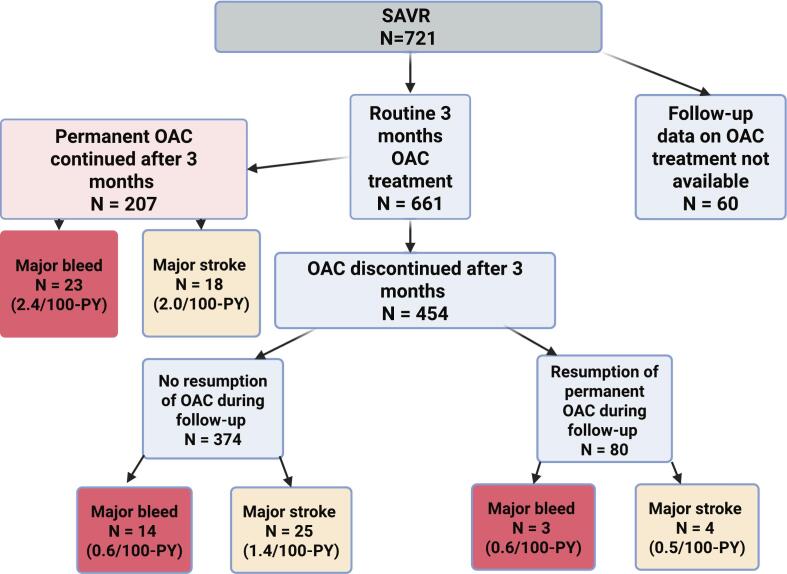
Flowchart of major bleeding events and their relations to oral anticoagulation (OAC) treatment three months after bioprosthetic isolated surgical aortic valve replacement (SAVR).

During the long-term follow-up period (more than 30 days after the index surgery), 40 major bleeding events (5.5 %) were recorded. The baseline characteristics of these patients are presented in Table 2. The event rate for major bleeding was 1.2 per 100 patient-years. The cumulative incidence of major bleeding at three months, 1, 2, and 5 years were 0.6 %, 1.0 %, 2.0 %, and 4.4 %, respectively (Fig. 2). In the 40 patients with major bleeding event, the most common type of bleeding was gastrointestinal (n = 17, 42.5 %), followed by intracranial (n = 14, 35.0 %). At the time of the first major bleeding event, 23 patients (57.5 %) were on OAC, of whom 22 (95.7 %) were using VKA. In patients with VKA treatment, 55 % of the bleeding events occurred when the INR value was within the target range (INR 2 to 3) (Fig. 3). In patients with permanent OAC continued directly after the routine three-month treatment, the event rate for major bleeding was 2.4 per 100 patient-years. Furthermore, in this patient cohort, the event rate for major stroke was 2.0 events per 100 patient-years. OAC was discontinued after the routine three-month treatment in 454 patients, while in 374 patients it was not re-initiated during follow-up. Among patients without permanent OAC during the follow-up, the event rate for major bleeding was 0.6 per 100 patient-years. In this patient cohort, the event rate for major stroke was 1.4 per 100 patient-years. Additionally, of the 80 patients who re-initiated OAC after discontinuation at three months, the event rate for major bleeding was 0.6 per 100 patient-years. Among patients who re-initiated OAC after discontinuation at three months, the median time to treatment resumption was 1.9 (IQR 0.4–4.4) years. The relationship between OAC and the cumulative incidence of major bleeding is illustrated in Fig. 4 and Fig. 5. During the long-term follow-up, 47 (6.5 %) patients experienced a major stroke. TIA occurred in 33 (4.6 %) patients, excluding patients with major stroke events. In addition, 22 (46.8 %) of the events occurred on OAC treatment. In the Cox proportional hazards model, permanent OAC after three months was associated with over twofold increased hazard of the combined event of major stroke or bleed (HR 2.44, 95 % CI 1.55–3.84, p < 0.001).

**Table 2:** Baseline characteristics and operative data of patients who underwent isolated bioprosthetic aortic valve replacement with and without major bleeding event after 30 days postoperatively.

	**Major bleed after 30 days (n = 40)**	**No Major bleed after 30 days** **(n = 681)**	**HR (95 % CI)**	**P-value**
Age	75.3 (72.7–77.8)	76.2 (71.7–80.0)	0.99 (0.94–1.04)	0.660
Females	23 (57.5 %)	384 (56.4 %)	1.04 (0.55–2.00)	0.900
Diabetes	10 (25.0 %)	132 (19.4 %)	1.98 (0.94–4.20)	0.071
Dyslipidemia	23 (57.5 %)	385 (56.7 %)	1.03 (0.54–1.98)	0.910
Hypertension	35 (87.5 %)	500 (73.5 %)	3.75 (1.26–11.3)	0.017
Coronary artery disease	13 (32.5 %)	177 (25.9 %)	1.36 (0.68–2.74)	0.390
Atrial fibrillation	16 (40.0 %)	167 (24.5 %)	2.26 (1.18–4.34)	0.014
Chronic atrial fibrillation	11 (27.5 %)	76 (11.6 %)	4.25 (2.06–8.81)	<0.001
Paroxysmal atrial fibrillation	5 (12.5 %)	91 (13.4 %)	0.76 (0.27–2.17)	0.610
Chronic lung disease	10 (25.0 %)	121 (17.8 %)	1.60 (0.75–3.42)	0.222
Active smoking	1 (3.3 %)	49 (8.0 %)	0.44 (0.06–3.35)	0.430
Active or ex-smoker	10 (38.5 %)	171 (29.4 %)	1.28 (0.54–3.00)	0.570
Body mass index (kg/m2)	26.6 (24.7–28.6)	27.6 (24.5–30.8)	1.00 (0.99–1.01)	0.780
Active endocarditis	2 (5.0 %)	16 (2.3 %)	1.93 (0.45–8.28)	0.380
Previous venous thromboembolism	1 (2.5 %)	16 (2.4 %)	0.95 (0.12–7.37)	0.960
Previous stroke or TIA	6 (5.8 %)	101 (15.5 %)	0.69 (0.26–1.82)	0.450
Previous myocardial infarction	4 (10.0 %)	46 (6.8 %)	1.59 (0.55–4.59)	0.390
Previous percutaneous coronary intervention	8 (20.0 %)	46 (6.8 %)	3.03 (1.26–7.28)	0.013
Previous cardiac surgery	1 (2.5 %)	38 (5.6 %)	0.48 (0.06–3.62)	0.480
EuroSCORE II (%)	2.1 (1.4–2.9)	1.7 (1.2–2.5)	1.08 (1.01–1.15)	0.019
NYHA Class III or more	24 (60.0 %)	342 (50.2 %)	1.25 (0.63–2.45)	0.520
Left ventricular ejection fraction (%)	60.0 (47.5–66.8)	60.0 (50.3–70.0)	0.98 (0.95–1.00)	0.110
Left atrium diameter (mm)	45.2 ± 6.9	43.0 ± 7.7	1.03 (0.99–1.07)	0.200
Aortic valve peak pressure gradient (mmHg)	83.0 (70.5–90.0)	79.0 (66.0–95.0)	0.99 (0.98–1.01)	0.290
Aortic valve regurgitation	30 (76.9 %)	364 (55.7 %)	2.24 (1.01–4.94)	0.046
Aortic valve regurgitation degree*	1.0 (1.0–2.5)	1.0 (1.0–1.0)	1.47 (1.03–2.09)	0.032
Mitral valve regurgitation	31 (77.5 %)	361 (54.8 %)	2.81 (1.27–6.19)	0.011
Mitral valve regurgitation degree†	1.0 (1.0–1.5)	1.0 (1.0–1.0)	2.39 (1.50–3.81)	<0.001
Pulmonary hypertension	18 (52.9 %)	172 (29.3 %)	2.90 (1.43–5.89)	0.003
Urgent, emergency or salvage procedure	3 (7.5 %)	30 (4.4 %)	2.67 (0.78–9.12)	0.120
Heart rate	73.0 (65.5–80.0)	68.0 (61.0–77.0)	1.01 (0.99–1.03)	0.280
Valve prosthesis size (mm)	23.0 (21.8–24.3)	23.0 (21.0–23.3)	1.00 (0.85–1.17)	0.970
Preoperative laboratory values:				
eGFR	69.5 ± 21.4	74.5 ± 21.2	0.99 (0.97–1.01)	0.300
Postoperative laboratory values:				
eGFR minimum	63.0 (46.3–86.5)	61.0 (48.0–79.0)	0.99 (0.97–1.00)	0.140
NOAF during index hospitalization	20 (50.0 %)	312 (46.1 %)	1.11 (0.59–2.10)	0.740
Cardioversion during hospitalization	4 (10.3 %)	116 (17.1 %)	0.45 (0.16–1.28)	0.130
Reoperation due to bleeding	3 (7.5 %)	59 (8.7 %)	0.64 (0.19–2.10)	0.460
Delayed ventilation	5 (12.5 %)	65 (9.6 %)	1.67 (0.65–4.3)	0.290
Acute de novo dialysis	1 (2.5 %)	11 (1.6 %)	7.30 (0.95–55.9)	0.056
Length of hospital stay (days)	8.5 (7.0–12.3)	8.0 (7.0–11.0)	1.04 (1.01–1.07)	0.001

Continuous variables are reported as median (25th – 75th percentiles) or mean ± standard deviation (SD). Values in parentheses are percentages. HR, Hazard Ratio; CI, Confidence Interval; eGFR: Estimated Glomerular Filtration Rate; EuroSCORE: European System for Cardiac Operative Risk Evaluation; INR: International Normalized Ratio; NOAF: New-onset Atrial Fibrillation; NYHA: New York Heart Association; TIA: Transient Ischemic Attack. * Within the patients with aortic valve regurgitation. † Within the patients with mitral valve regurgitation.

In the multivariable mixed effect analysis, the independent predictors of major bleeding during the long-term follow-up were preoperative hypertension (HR 3.75, 95 % CI 1.26–11.3, p = 0.017), preoperative AF (HR 2.26, 95 % CI 1.18–4.34, p = 0.014), previous percutaneous coronary intervention (PCI) (HR 3.03, 95 % CI 1.26–7.28, p = 0.013), higher EuroSCORE II (HR 1.08, 95 % CI 1.01–1.15, p = 0.019 per one percent increment), higher aortic valve regurgitation (AR) degree (HR 1.47, 95 % CI 1.03–2.09, p = 0.032), higher mitral valve regurgitation (MR) degree (HR 2.39, 95 % CI 1.50–3.81, p < 0.001) and pulmonary hypertension (HR 2.90, 95 % CI 1.43–5.89, p = 0.003). Major bleeds were associated with longer index hospital stay (HR 1.04, 95 % CI 1.01–1.07, p = 0.001). Graphical assessment of the scaled Schoenfeld residuals indicated no violations of the proportional hazards assumption for any of the covariates.

## 4 Discussion

The major take home point of the present study is that during the postoperative 30-day period, the risk of major bleeding was twice that of major stroke. Nearly a half of the bleedings occurred within a couple of days after surgery – often during the tail effect of preoperatively used ASA. In the long-term follow-up, the risks of stroke and bleeding were nearly identical, with approximately 1 in 20 patients in both groups experiencing either a major bleeding event or a major stroke. Furthermore, most late bleeding episodes occurred while the patients were on anticoagulation treatment.

### 4.1 Early bleeding and stroke under antithrombotic therapy

Despite abundance of prior reports on the occurrence of postoperative complications after bioprosthetic SAVR, there is a lack of data concerning the timing of antithrombotic treatment in relation to these complications [11], [12], [13], [14]. This study provided detailed day-to-day information regarding short-term antithrombotic therapy, which facilitated a more nuanced understanding of early postoperative complications than has been previously reported. During the postoperative 30-day period, the incidence of major bleeding was significantly higher than that of early major strokes. Most bleeding events occurred early following the surgical procedure, with INR typically below the therapeutic range. However, early postoperative bleeding must be interpreted primarily in the context of the surgical intervention itself. The immediate post-surgical period involves tissue injury, inflammatory activation, and coagulopathy induced by cardiopulmonary bypass or surgical trauma, all of which substantially increase bleeding susceptibility independent of antiplatelet therapy. The residual antiplatelet effect of ASA can potentiate these challenges. Importantly, this setting represents the early, subtherapeutic phase of VKA initiation and should not be interpreted as a true pharmacological triple therapy, but a setting where many components of coagulation cascade are in unpredictable state. During VKA initiation, protein C levels decline rapidly while procoagulant factors decrease more slowly, creating a transient imbalance favouring thrombosis early in therapy [15], [16]. Considering that ASAs pharmacological effects may persist for up to 7 days [15], many observed incidences occurred in this context, where residual ASA activity, enoxaparin therapy, and subtherapeutic VKA overlapped. In addition, the event rate for major bleeding was higher in patients with ASA tail effect during the surgery than in patients without such effect. The current literature remains limited regarding the optimal combination of preoperative and perioperative antithrombotic therapies balancing between bleeding and ischemic event risk in patients undergoing SAVR. In patients undergoing bioprosthetic SAVR, it has been demonstrated that VKA and ASA exhibit comparable antithrombotic efficacy in stroke prevention [17]. The results of our study raise a hypothesis that the ASA tail effect may increase the risk of major bleeding, and adequate withdrawal of preoperative ASA could reduce the risk of early perioperative bleeding. However, these findings must be verified in a larger scale prospective randomized setting with current anticoagulation strategies.

### 4.2 Incidence and predictors of early bleeding and stroke

In previous studies, the incidence of 30-day postoperative major bleeding has varied considerably, ranging from 1.8 % up to 43.4.% [11], [14], [18], [19]. The observed differences in bleeding rates are likely attributable to differences in patient populations and variations in major bleeding definitions across studies. Standardizing of definitions would enable more accurate comparisons of bleeding events.

Preoperative use of ADP receptor inhibitors was also identified as a predictor of early postoperative bleeding, and bleeding events were more frequent among patients receiving ADP receptor inhibitors. ADP receptor inhibitors are a known risk factor for major bleeding [20]. The risk was particularly pronounced in those who are also treated with ASA, indicating that quadruple therapy is especially hazardous. Moreover, in patients receiving PPIs during hospital discharge, the increased risk of bleeding is likely due to in-hospital post-operative bleeding events; PPIs have been initiated to prevent the recurrence of bleeding events. Additionally, patients who experienced an early postoperative major stroke were more likely to be discharged on ASA therapy. This difference likely reflects clinical response to the cerebrovascular event rather than pre-existing factors that predisposed patients to stroke. We emphasize that this treatment pattern is better interpreted as a consequence of the stroke rather than its predictors.

### 4.3 Incidence and predictors of long-term bleeding and stroke

During the long-term follow-up, approximately 1 out of 20 patients experienced a major bleeding event, with similar proportion experiencing a major stroke. A detailed analysis of the long-term cerebrovascular events in the present cohort has been reported in a previous study [7]. In earlier studies, the incidence on long-term major bleeding has varied, ranging from 4.0 % up to 47.0 % [1], [11], [12], [21], [22]. However, many of these studies include 30-day bleeding events in the long-term bleeding data, resulting in higher incidence rates than what is observed in reality. In the current study, the leading cause of major bleeds was gastrointestinal bleeding, accounting for nearly half of the total incidents. Similar findings have been reported in previous studies [12], [23].

Hypertension, VKA treatment, and pulmonary hypertension are commonly recognized predictors of bleeding [24], [25]. The effect of preoperative AF is likely due to the permanent need for OAC therapy. Although patients with aortic stenosis are known to have a higher risk of bleeding due to Heyde’s syndrome [26], no direct connection between AR or MR and bleeding is known. One potential reason for the finding is that heart failure is relatively common in patients with AR or MR, and it is known to increase the risk of bleeding [27]. Moreover, heart failure is also a common risk factor for AF [28].

### 4.4 Impact of antithrombotic therapy on long-term bleeding and stroke

Most major bleeding events in long-term follow-up occurred during OAC treatment – mostly with VKA. The high proportion of VKA is explained by the fact that non-vitamin K oral anticoagulants (NOAC) were infrequently used at the time of the study. Although most major bleeding events occurred when the INR value was within the therapeutic range in VKA-treated patients, a notable proportion of the events occurred when the INR target was out of range. In a recent FINACAF study, therapeutic target range (TTR) values > 80 were associated with lower rates of both bleeding and strokes, underscoring the need for careful monitoring of VKA treatment [29], [8]. The American guideline assigns a Class IIb and the European guideline a Class IIa recommendation to the use of warfarin for three months following bioprosthetic SAVR, indicating that OAC may or should be considered during this period. The European guideline further allows for the use of ASA instead of OAC in patients without another indication for anticoagulation based on the patient's bleeding risk profile [3], [30]. In the current study, OAC was discontinued after the routine three-month treatment in two thirds of patients. Among patients with permanent OAC continued immediately after the routine three-month treatment, 1 in 10 experienced major bleeding, compared to less than 1 in 20 among those with OAC discontinued after three months. In addition, patients who remained on permanent OAC after three months experienced a significantly higher risk of the combined endpoint (major bleeding or ischemic stroke occurring > 30 days postoperatively) compared with those whom OAC discontinued after the three-month period. Furthermore, in this patient cohort, the yearly event rates for bleeding and stroke were nearly equal, suggesting a similar balance of risks in this subgroup. While OAC therapy is essential when there is a clear indication for anticoagulation, these findings support the view to avoid OAC use in borderline indications, such as perioperative or subclinical AF, due to the high bleeding risk of the population. Additionally, potential preventive strategies, such as long-term PPI therapy, may be of benefit when used more often.

## 5 Study limitations

The main limitation of this study is the retrospective nature of the CAREAVR data. However, the data were obtained from electronic patient records, and data on baseline, operation, and outcomes are reported in detail at each of the participating hospitals. Only patients from the hospitals’ catchment areas were included in this study to obtain reliable and accurate follow-up data. Also, the follow-up was complete for as much as 99.7 % of the patients as the treatment of patients belonging to the catchment areas of the participating institutions is mainly centralized. This allowed us to obtain information on major clinical events requiring hospital treatment and information on outpatient visits after the surgery because they were performed almost exclusively in tertiary health care. As a quality control of the multicenter CAREAVR database, a professional third party monitored the data. However, the study did not include independent adjudication of clinical events, which may introduce potential bias in event classification. Data on late mortality were obtained from the Statistics Finland, which ensures the quality of survival data of the patients. Another limitation is that the data is over a decade old and might not reflect current anticoagulation strategies. As our study was conducted only in one country, our results might not reflect those of other populations. In addition, the early outcomes analysis is based on perioperative data from a single center, which may affect generalizability. Our relatively small sample size is a limitation, and therefore, these findings should be viewed as hypothesis-generating.

## 6 Conclusions

In conclusion, after bioprosthetic SAVR, the incidence of early postoperative major bleeding is more than twice that of major stroke, with most bleeding events occurring during the tail effect of preoperative ASA. During long-term follow-up, the risks of stroke and bleeding are nearly equal, with most bleeding episodes occurring while patients are on anticoagulation treatment.

## Ethical approval

The study protocol (ETMK:8/1802/2014) was approved by the Medical Ethics Committee of the Hospital District of Southwest Finland and the ethics committee of the National Institute for Health and Welfare (Finland) (T39/2015). Because of the retrospective, observational nature of the study, informed consent was not required. The study conformed to the Declaration of Helsinki as revised in 2002.

## CRediT authorship contribution statement

**Rikhard Björn:** Writing – review & editing, Writing – original draft, Methodology, Investigation, Formal analysis. **Joonas Lehto:** Writing – review & editing, Methodology, Investigation, Formal analysis. **Markus Malmberg:** Writing – review & editing. **Vesa Anttila:** Writing – review & editing. **Jarmo Gunn:** Writing – review & editing. **Tuomo Nieminen:** Writing – review & editing. **Juha E.K. Hartikainen:** Writing – review & editing. **Fausto Biancari:** Writing – review & editing. **K.E.Juhani Airaksinen:** Writing – review & editing. **Tuomas Kiviniemi:** Writing – review & editing, Supervision, Resources, Project administration, Funding acquisition, Data curation.

## Declaration of competing interest

The authors declare that they have no known competing financial interests or personal relationships that could have appeared to influence the work reported in this paper.
