# Supplementary material for: Major bleeding complications and antithrombotic treatment after isolated surgical bioprosthetic aortic valve replacement
Source: Int J Cardiol Heart Vasc. 2026 Jan 6;62:101861. doi: 10.1016/j.ijcha.2025.101861 (PMC13153142; doi:10.1016/j.ijcha.2025.101861)
Supplement: Supplementary Data 1 [file mmc1.docx]

**Supplementary Table 1.** Baseline characteristics, operative data, and long-term outcomes of patients in the 227-patient subgroup and the remaining 494 patients in the patient population.

|  | **Subgroup, n=227** | **Rest of the patient population,  n=494** | **p value** |
| --- | --- | --- | --- |
| Age | 77.2 ± 3.9 | 75.2 ± 7.6 | <0.001 |
| Females | 129 (56.8%) | 278 (56.3%) | 0.889 |
| Diabetes | 38 (16.8%) | 104 (21.1%) | 0.185 |
| Dyslipidemia | 128 (56.6%) | 280 (56.8%) | 0.968 |
| Hypertension | 188 (82.2%) | 347 (70.4%) | 0.001 |
| Coronary artery disease | 95 (41.9%) | 95 (19.2%) | <0.001 |
| Atrial fibrillation | 54 (23.8%) | 129 (26.1%) | 0.505 |
| Chonic atrial fibrillation | 29 (12.8%) | 58 (11.7%) | 0.692 |
| Paroxysmal atrial fibrillation | 25 (11.0%) | 71 (14.4%) | 0.218 |
| Chronic lung disease | 32 (14.1%) | 99 (20.2%) | 0.050 |
| Active smoking | 12 (7.4%) | 38 (7.9%) | 0.824 |
| Active or ex-smoker | 59 (37.8%) | 122 (27.1%) | 0.113 |
| Body mass index (kg/m2) | 27.5 (24.5–29.7) | 27.6 (24.2–21.1) | 0.328 |
| Active endocarditis | 6 (2.6%) | 12 (2.4%) | 0.867 |
| Previous endocarditis | 3 (1.3%) | 5 (1.0%) | 0.717 |
| Previous venous thromboembolism | 4 (1.7%) | 13 (2.6%) | 0.473 |
| Previous stroke or TIA | 36 (15.9%) | 71 (15.4%) | 0.867 |
| Previous myocardial infarction | 14 (6.2%) | 36 (7.3%) | 0.573 |
| Previous percutaneous coronary intervention | 15 (6.6%) | 39 (7.9%) | 0.537 |
| Previous cardiac surgery | 7 (3.1%) | 32 (6.5%) | 0.061 |
| EuroSCORE II (%) | 1.7 (1.4–2.6) | 1.7 (1.2–2.4) | 0.718 |
| NYHA Class III or more | 153 (67.4%) | 213 (43.1%) | <0.001 |
| NOAF during index hospitalization | 111 (48.9%) | 221 (45.1%) | 0.343 |
| Cardioversion during hospitalization | 66 (29.3%) | 54 (11.0%) | <0.001 |
| Acute de novo dialysis | 1 (0.4%) | 11 (2.2%) | 0.080 |
| Length of hospital stay | 8.0 (6.0–9.0) | 9.0 (7.3–13.0) | <0.001 |
| **Echocardiographic parameters:** |  |  |  |
| Valve prosthesis size (mm) | 23.0 (21.0–23.0) | 23.0 (22.0–25.0) | 0.331 |
| Left ventricular ejection fraction (%) | 61.0 (54.0–70.0) | 60.0 (50.0–68.0) | 0.076 |
| Aortic valve regurgitation | 143 (65.3%) | 251 (53.0%) | 0.002 |
| Aortic valve peak pressure gradient (mmHg) | 60.7 ± 13.0 | 59.1 ± 12.1 | 0.144 |
| Mitral valve regurgitation | 149 (67.4%) | 243 (50.8%) | <0.001 |
| Pulmonary hypertension | 66 (40.9%) | 124 (26.9%) | <0.001 |
| **Long-term outcomes:** |  |  |  |
| Major bleed | 22 (9.7%) | 18 (3.6%) | 0.001 |
| Major stroke | 25 (11.0%) | 22 (4.5%) | 0.001 |

Values in parentheses are percentages. EuroSCORE: European System for Cardiac Operative Risk Evaluation; NOAF: New-onset Atrial Fibrillation; NYHA: New York Heart Association; TIA: Transient Ischemic Attack.
