# Supplementary material for: Major bleeding complications and antithrombotic treatment after isolated surgical bioprosthetic aortic valve replacement
Source: Int J Cardiol Heart Vasc. 2026 Jan 6;62:101861. doi: 10.1016/j.ijcha.2025.101861 (PMC13153142; doi:10.1016/j.ijcha.2025.101861)
Supplement: Supplementary Data 2 [file mmc2.docx]

**Supplementary Table 2.** Baseline characteristics and operative data for the 227-patient subgroup, stratified by preoperative ASA use.

|  | **With preoperative ASA, n=138** | **Without preoperative ASA,  n=89** | **p value** |
| --- | --- | --- | --- |
| Age | 77.1 ± 4.0 | 77.0 ± 3.7 | 0.782 |
| Females | 62 (44.9%) | 36 (40.4%) | 0.755 |
| Diabetes | 27 (19.6) | 11 (12.4) | 0.205 |
| Dyslipidemia | 90 (65.2%) | 38 (42.7%) | 0.003 |
| Hypertension | 119 (86.2%) | 69 (77.5%) | 0.420 |
| Coronary artery disease | 65 (47.1%) | 30 (33.7%) | 0.097 |
| Atrial fibrillation | 20 (14.5%) | 34 (38.2%) | <0.001 |
| Chonic atrial fibrillation | 4 (2.9%) | 25 (28.1%) | <0.001 |
| Paroxysmal atrial fibrillation | 16 (11.6%) | 9 (10.1%) | 0.837 |
| Chronic lung disease | 19 (13.8%) | 13 (14.6%) | 0.730 |
| Active smoking | 6 (5.6%) | 6 (10.7%) | 0.236 |
| Active or ex-smoker | 39 (37.5%) | 20 (38.5%) | 0.907 |
| Body mass index (kg/m2) | 27.2 (± 4.7) | 28.1 (± 4.4) | 0.203 |
| Active endocarditis | 2 (1.4%) | 4 (4.5%) | 0.141 |
| Previous endocarditis | 3 (2.2%) | 0 (0.0%) | 0.173 |
| Previous venous thromboembolism | 1 (0.7%) | 3 (3.4%) | 0.123 |
| Previous stroke or TIA | 28 (20.3%) | 8 (9.0%) | 0.030 |
| Previous myocardial infarction | 13 (9.4%) | 1 (1.1%) | 0.144 |
| Previous percutaneous coronary intervention | 12 (8.7%) | 3 (3.4%) | 0.140 |
| Previous cardiac surgery | 4 (2.9%) | 3 (3.4%) | 0.783 |
| EuroSCORE II (%) | 1.7 (1.4–2.5) | 1.8 (1.4–3.0) | 0.070 |
| NYHA Class III or more | 93 (67.4%) | 60 (67.4%) | 0.552 |
| NOAF during index hospitalization | 44 (31.7%) | 22 (25.6%) | 0.331 |
| Cardioversion during hospitalization | 21 (15.2%) | 9 (10.0%) | 0.339 |
| Acute de novo dialysis | 1 (0.7%) | 0 (2.2%) | 0.433 |
| Length of hospital stay | 8.0 (6.0–9.0) | 8.0 (6.3–9.0) | 0.860 |
| **Echocardiographic parameters:** |  |  |  |
| Left ventricular ejection fraction (%) | 61.0 (54.3–70.0) | 62.0 (54.0–70.0) | 0.549 |
| Aortic valve regurgitation | 89 (65.0%) | 54 (65.9%) | 0.893 |
| Aortic valve peak pressure gradient (mmHg) | 88.7 ± 22.6 | 88.5 ± 22.7 | 0.965 |
| Mitral valve regurgitation | 88 (63.8%) | 61 (74.4%) | 0.090 |
| Pulmonary hypertension | 29 (30.9%) | 37 (55.2%) | 0.002 |

Values in parentheses are percentages. ASA: Acetylsalicylic Acid; EuroSCORE: European System for Cardiac Operative Risk Evaluation; NOAF: New-onset Atrial Fibrillation; NYHA: New York Heart Association; TIA: Transient Ischemic Attack.
